# Examining the Effect of Transcranial Electrical Stimulation and Cognitive Training on Processing Speed in Pediatric Attention Deficit Hyperactivity Disorder: A Pilot Study

**DOI:** 10.3389/fnhum.2022.791478

**Published:** 2022-07-27

**Authors:** Ornella Dakwar-Kawar, Itai Berger, Snir Barzilay, Ephraim S. Grossman, Roi Cohen Kadosh, Mor Nahum

**Affiliations:** ^1^School of Occupational Therapy, Faculty of Medicine, The Hebrew University of Jerusalem, Jerusalem, Israel; ^2^Pediatric Neurology, Assuta-Ashdod University Medical Center, Faculty of Health Sciences, Ben-Gurion University of the Negev, Be’er Sheva, Israel; ^3^Paul Baerwald School of Social Work and Social Welfare, Hebrew University, Jerusalem, Israel; ^4^School of Psychology, Faculty of Health and Medical Sciences, University of Surrey, Guildford, United Kingdom

**Keywords:** ADHD, CPT, processing speed, inhibitory control, transcranial electrical stimulation, tRNS, tDCS, cognitive fatigue

## Abstract

**Objective:**

Processing Speed (PS), the ability to perceive and react fast to stimuli in the environment, has been shown to be impaired in children with attention deficit hyperactivity disorder (ADHD). However, it is unclear whether PS can be improved following targeted treatments for ADHD. Here we examined potential changes in PS following application of transcranial electric stimulation (tES) combined with cognitive training (CT) in children with ADHD. Specifically, we examined changes in PS in the presence of different conditions of mental fatigue.

**Methods:**

We used a randomized double-blind active-controlled crossover study of 19 unmedicated children with ADHD. Participants received either anodal transcranial direct current stimulation (tDCS) over the left dorsolateral prefrontal cortex (dlPFC) or transcranial random noise stimulation (tRNS), while completing CT, and the administration order was counterbalanced. PS was assessed before and after treatment using the MOXO-CPT, which measures PS in the presence of various conditions of mental fatigue and cognitive load.

**Results:**

tRNS combined with CT yielded larger improvements in PS compared to tDCS combined with CT, mainly under condition of increased mental fatigue. Further improvements in PS were also seen in a 1-week follow up testing.

**Conclusion:**

This study provides initial support for the efficacy of tRNS combined with CT in improving PS in the presence of mental fatigue in pediatric ADHD.

## Introduction

Attention-deficit/hyperactivity disorder (ADHD) is the most common neuro-developmental disorder in childhood, affecting 5–9% of school-aged children (American Psychiatric Association, 2013; Danielson et al., 2018). Symptoms of ADHD include inattention, hyperactivity, and impulsivity (American Psychiatric Association, 2013; Visser et al., 2014). ADHD is not just a childhood disorder; since up to two thirds of children who were diagnosed with ADHD still meet ADHD criteria in adulthood, showing long-term negative effects (Karam et al., 2015).

Importantly, processing speed (PS), a fundamental cognitive ability which measures the speed at which one can perceive and react to stimuli in the environment (Fry and Hale, 2000; Peled et al., 2020), has been suggested to be abnormally low in ADHD (Barkley et al., 1990; Barkley et al., 1992; Hartman et al., 2004; Mayes et al., 2009; Brown, 2013; Moore and Ledbetter, 2019). However, despite the proliferation of research associating pediatric ADHD with reduction in PS (Cook et al., 2018), there is currently no consensus definitions of PS as a neuropsychological construct in ADHD (Shanahan et al., 2006). Some studies have operationalized PS using reaction time (RT) measurement, i.e., the time one takes to complete simple cognitive tasks (Fry and Hale, 2000). However, it has been suggested that this definition of PS is too board, and does not take into account the balance between speed and accuracy (Rommelse et al., 2020). Other studies referred to PS as the ability to perform correctly and accurately on a cognitive task within a given time window. An example for this type of measure is given by the Processing Speed Index (PSI) from the Wechsler intelligence test, which measures the ability to quickly and correctly discriminate simple visual information (Hedvall et al., 2013; Sherwell et al., 2014; Cook et al., 2018). Similarly, the PS (timing) metric of MOXO-CPT measures the number of correct responses given while the target stimulus is still presented on the screen (Elbaum et al., 2020; Peled et al., 2020).

Using continuous performance test (CPT) designs, several studies have reported slower and more variable PS in children with ADHD compared to matched neurotypical children (van der Meere et al., 1995; Rubia et al., 1998; Nigg, 2001; Scheres et al., 2001; Slusarek et al., 2001; Rucklidge and Tannock, 2002; Willcutt et al., 2005; Shanahan et al., 2006; Kofler et al., 2013; Westwood et al., 2021). Moreover, PS is considered by some authors as one of the best predictors of inattentive symptoms in ADHD (Chhabildas et al., 2001; Weiler et al., 2001; Rucklidge and Tannock, 2002). Weigard and Huang-Pollock (2017) suggested that slow PS is a plausible cause for the working memory (WM) deficits associated with ADHD. The cognitive-energetic hypothesis of ADHD further postulates that children with ADHD are cognitively under-aroused and thus have slower and more variable PS (van der Meere et al., 1995; Castellanos et al., 2005; Sergeant, 2005; Shanahan et al., 2006; Weigard and Huang-Pollock, 2017). Interestingly, PS is further reduced in ADHD compared to neurotypical controls when there is an increased load of auditory or visual distractors during a CPT task. Specifically, while for neurotypical children only a combination of visual and auditory distractors created enough cognitive load to impair their attention, for children with ADHD a lower load generated by either auditory or visual distractors impaired their performance in the task (Cassuto et al., 2013). This finding that can be accounted for by the inhibitory control deficits and difficulty in filtering out irrelevant distractors in ADHD (Blakeman, 2000; Cassuto et al., 2013).

In addition to distractibility as a cognitive deficit that may affect performance in a CPT, cognitive fatigue over time, which is induced by prolonged cognitive load (Mizuno et al., 2011) or by prolonged periods of cognitive activity (Boksem et al., 2005; DeLuca, 2018), may also lead to reduced PS and sustained attention in ADHD (Holtzer et al., 2011; Bioulac et al., 2012; Slobodin et al., 2020; Ayache and Chalah, 2021). Moreover, children with ADHD are more prone to show reduced performance during a long battery of cognitive tasks compared to their neurotypical peers due to cognitive fatigue; these effects are consistent with their PS deficits over time during cognitive tasks (McGee et al., 2004; Pelham et al., 2011; Bioulac et al., 2012; Slobodin et al., 2020). Despite the significance of PS as a therapeutic target in ADHD, studies applying various forms of interventions such as cognitive training (Adalio et al., 2018; Moore and Ledbetter, 2019; Kollins et al., 2020), neurofeedback (Bink et al., 2014), psychopharmacological treatments (Graziano et al., 2010; Nielsen and Wiig, 2011; Adalio et al., 2018; Peled et al., 2020), and non-invasive brain stimulations (Looi et al., 2017; Brevet-Aeby et al., 2019; Harty and Cohen Kadosh, 2019; Berger et al., 2021; Westwood et al., 2021), yielded mixed results. In addition, little is known about the effects of these treatments on PS in the presence of cognitive fatigue or when environmental distractions are present.

In the current study, we examined possible changes in PS in children with ADHD following an experimental therapeutic approach, which included non-invasive transcranial electric stimulation (tES) performed simultaneously with cognitive training (CT). We contrasted two forms of tES: transcranial direct current stimulation (tDCS) and transcranial random noise stimulation (tRNS), both involve the passage of a weak electrical current through one or more electrodes placed on the scalp (Polanía et al., 2018; Reed and Cohen Kadosh, 2018). While anodal tDCS increases cortical excitability, cathodal stimulation decreases it (Polanía et al., 2018). tDCS has shown some promise as a potential treatment avenue for neuropsychological deficits (Krause and Cohen Kadosh, 2013; Santarnecchi et al., 2015; Faundez et al., 2018; Rubia, 2018), and particularly in ameliorating symptoms and EFs in children with ADHD (Soltaninejad et al., 2015; Looi et al., 2017; Sotnikova et al., 2017; Zhao et al., 2017; Allenby et al., 2018; Rubia, 2018), specifically when combined with CT (Rubia, 2018). Interestingly, while tDCS was shown to improve inhibitory control accuracy, it did not affect PS in a Go-NoGo (GNG) task (Allenby et al., 2018; Salehinejad et al., 2019; Thunberg et al., 2020; Westwood et al., 2021). However, no systematic evaluation of task-specific protocols has been conducted for the effects of tDCS on PS thus far (Kuo and Nitsche, 2015; Nejati et al., 2017; Salehinejad et al., 2019).

tRNS, on the other hand, is a form of tES that uses the same electrode composition as tDCS to stimulate neuronal activity, but with both electrodes being used to increase cortical excitability (Terney et al., 2008). It has been suggested that the effect of tRNS on task performance is amplified when the load demands of the task are increased (Popescu et al., 2016; Sheffield et al., 2020). Only a few studies to date examined the effects of tRNS on PS, showing mixed results (Looi et al., 2017; Brevet-Aeby et al., 2019; Harty and Cohen Kadosh, 2019; Berger et al., 2021). For example, a study in healthy adults demonstrated long-term improvements in PS following tRNS, as measured by decreased RTs for “Go” trials in a GNG task (Brevet-Aeby et al., 2019). However, another study failed to find an overall effect of tRNS on PS measured in a sustained attention CPT, but instead reported reduced intra-subject variability in PS following tRNS in neurotypical adults (Harty and Cohen Kadosh, 2019). A study by Looi et al. (2017), which examined the effect of tRNS combined with CT on children with mathematical learning disabilities, found that the combined treatment improved learning and performance during arithmetic training tasks, but did not affect PS (Looi et al., 2017).

In a recently published manuscript (Berger et al., 2021) we found that tRNS combined with CT yields significantly larger improvements in clinical symptoms, WM and PS compared to tDCS + CT. The aim of the current study was to systematically map changes in PS following tES (tDCS vs. tRNS) combined with CT in a pediatric ADHD sample, in the presence of environmental distractions (visual and auditory) at various levels of load and fatigue. We hypothesized PS scores will be better following tRNS + CT compared to tDCS + CT with increased load (Popescu et al., 2016; Sheffield et al., 2020). We further asked whether tRNS + CT will also show improved PS in the presence of high levels of fatigue compared to tDCS + CT.

## Materials and Methods

### Study Design

**FIGURE 1:**
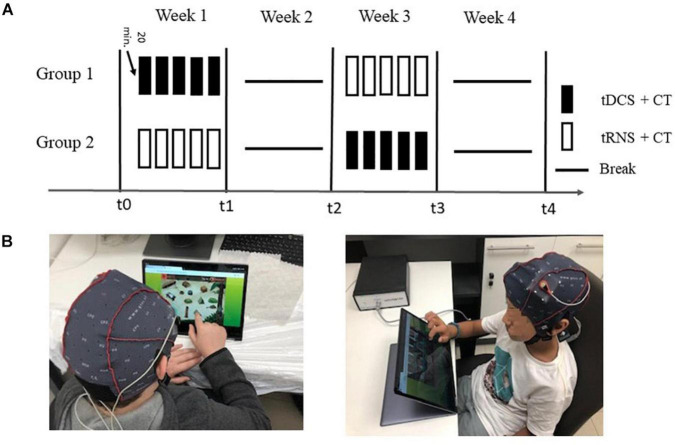
Study design. **(A)** Participants were randomized into two groups, each receiving treatment in weeks 1 and 3, with a gap period of no-treatment in weeks 2 and 4. Group 1 received tDCS + CT (black rectangles) in week 1 and transcranial random noise stimulation (tRNS) + CT (white rectangles) in week 3, while group 2 received the opposite pattern. PS was assessed at five time points during the study: baseline (t0) immediately post-treatment (t1, N-t1), and at follow-up, after a week of no treatment (t2, N-t2). **(B)** An example of tES + CT sessions. Children were seated in front of the tablet which delivered the CT, while being stimulated for 20 min each session. Pictures of children are included with written permission from participants and their parents.

Study design is depicted in Figure 1. The full study design was published elsewhere (Berger et al., 2021). Here we report the results of the outcomes related to PS. All study-related activities were conducted in the Computerized Neurotherapy lab at the School of Occupational Therapy of the Hebrew University of Jerusalem, Israel. The study was approved by the Helsinki Committee of the Hebrew University and Hadassah Medical Center (Jerusalem, Israel). The study is registered at ClinicalTrials.gov (identifier NCT03104972).

In a survey, 19 children diagnosed with ADHD (age: 6–12 y/o) participated in a randomized, controlled, double-blind crossover study. Following screening, eligible participants completed a battery of assessments at baseline and were then randomized into one of two treatment groups, each receiving tES combined with CT, but in different order. Participants in Group 1 (*n* = 10) first received tDCS + CT and then tRNS + CT, while participants in Group 2 (*n* = 9) received tRNS + CT and then tDCS + CT. Both groups received their designated treatment during week 1 and following a 1-week break on week 2, crossed-over to the other treatment on week 3. Participants remained blinded to the type of tES they received throughout the study. Since tDCS and tRNS have a different montage and to guarantee blindness, we alternated three naive research assistants to the protocol throughout this study. Both researchers and research assistants were also naïve to the randomization procedure, which assigned participants to a treatment group using Smith’s randomization algorithm (Good, 2006). This procedure was used for randomization as it is designed to generate balanced random samples throughout the course of an experiment.

To allow for an accurate assessment of the new treatment on week 3, we recalibrated the participants’ baseline measures by using their latest assessment data from t2 (1 week follow-up). A “new” baseline measure was taken at the beginning of week 3 (N-t0), and participants were assessed again at the end of week 3 (post-treatment; N-t1, N-post-treatment). At week 4 no treatment was given and the lasting effects from week 3 were measured at the end of week 4 (follow up; N-t2, N-FU).

Processing speed was measured at baseline (t0, N-t0) and immediately post-treatment (t1, N-t1) and was further assessed at follow up, at the end of each break-week (t2, N-t2), allowing us to assess lasting effects after 1 week of no treatment using a within-participant design. The total duration of participant participation in the study was 5 weeks, from screening until the last assessment visit.

### Study Population

Participants were recruited among children referred to an ADHD clinic by pediatricians, general practitioners, teachers, psychologists, or parents. In a survey, 34 children aged 6–12 y/o were assessed for eligibility, and of them, 21 passed screening and qualified to participate in the study. Out of 21 participants, 19 completed the study and two participants were dropped out from the study: one due to complaints of an uncomfortable topical sensation and headaches during the tDCS protocol and another due to parents reporting in the third session of behavior that might meet one of the exclusion criteria (the expression of self-harm thoughts, which was present already 2 months before study participation but was not reported at screening). A CONSORT diagram of the study is given in Supplementary Figure S1.

Inclusion criteria for participation were: (1) 6–12 years old; (2) meeting criteria for ADHD according to DSM-5 (American Psychiatric Association, 2013), using the “gold standard” procedure as described by the American Academy of Pediatrics, and including a semi-structured interview of the patient and parents by a specialist in pediatric neurology and child development, a neurological examination, and ADHD rating scale (ADHD-RS) diagnostic questionnaires (DuPaul et al., 1998; DuPaul et al., 2016). All children in the study scored above the standard clinical cutoff values for ADHD symptoms on ADHD DSM-5 scales (DuPaul et al., 1998, 2016; American Psychiatric Association, 2013); (3) New diagnosis of ADHD; (4) drug naïve.

Children were excluded from the study if they had one of the following: a chronic neurological disease, epilepsy in the participant or in a first-degree relative, intellectual disability, other chronic conditions, chronic use of medications, or other primary psychiatric diagnosis (e.g., depression, anxiety, psychosis). The Hebrew translation of the Kiddie-SADS-Lifetime Version (Kaufman et al., 2000) was used to assess Axis-I disorders in participants according to DSM-5 criteria (Kaufman et al., 2000). Prospective resting-state electroencephalography (EEG) was performed at screening to rule out an unknown existence of epileptiform activity. EEG records were standardized and recorded using the g.Recorder software (gTech, Schiedlberg, Austria) using a 32-channel wireless electroencephalography cap system (g.Nautilus) with gel-based electrodes.

### Study Interventions

Study interventions included tES (either tRNS or tDCS) applied simultaneously with CT. Group 1 participants received tRNS + CT on week 1 and tCDS + CT on week 3, while Group 2 participants received tRNS on week 1 and tDCS on week 3. Each treatment week comprised of five consecutive treatment days, with each daily treatment session lasting for 20 min. Thus, participants received a total of 100 min of training with each stimulation type (tRNS/tDCS) combined with CT during each treatment week, for a total of 200 min of stimulation and CT during the trial.

#### Transcranial Electrical Stimulation

Both tDCS and tRNS were applied using semi-dry 5 × 5 cm electrodes using the NovoStim device (Tech InnoSphere Eng. Ltd., Haifa, Israel).

##### Transcranial Direct Current Stimulation

The current was set to 0.75 mA based on previous computational modeling of tDCS in children and is estimated to equal that of approximately 1.5 mA in adults (Kessler et al., 2013). Ramp-up and ramp-down durations were 30 s each. These durations were chosen after considering the parameters that would influence current distribution and density at the site of stimulation, such as thinner scalp, less cerebrospinal fluid, and smaller head size of the pediatric population (Kessler et al., 2013). A similar dosage of tDCS was well tolerated by children and was not associated with adverse effects in previous studies (Krishnan et al., 2015). The anodal electrode was positioned above the left DLPFC (F3 based on the International 10-20 system), while the cathodal electrode was placed over the right supraorbital (Fp2). This montage has been deemed to be the most successful so far based on a meta-analysis of tDCS studies in ADHD (Salehinejad et al., 2019; Brauer et al., 2021).

##### Transcranial Random Noise Stimulation

Stimulation was applied at an amplitude of 0.75 mA of tRNS over the left DLPFC and the right inferior frontal gyrus (IFG), attached under designated electrode positions (F3-F8 based on the International 10-20 system) of the tES cap. These stimulation locations were chosen based on their involvement in executive control and inhibition processes (Castellanos et al., 2002; Aron et al., 2004; Christakou et al., 2004) and based on previous tRNS studies in the field of cognitive training in healthy young adults and children with dyscalculia (Snowball et al., 2013; Looi et al., 2017). Ramp-up and ramp-down durations were the same as in the tDCS condition.

#### Computerized Cognitive Training

Full details of the CT are given in a previous manuscript (Berger et al., 2021). In short, CT was comprised of four exercises from the ACTIVATE™ suite (Wexler et al., 2016), collectively targeting the EF functions of sustained attention, response inhibition, spatial working memory, cognitive flexibility, and switching. Each training session included four exercises, each played for 5 min, for a total duration of 20 min of gameplay per session, which coincided with the tES protocol.

The ACTIVATE™ cognitive training has been used in previous studies that aimed to improve academic performance in typically developing children (Wexler et al., 2016), and in some preliminary studies in children with ADHD (de Oliveira Rosa et al., 2020). For further details see Wexler et al. (2016).

### Processing Speed Assessment (MOXO-CPT)

The full list of outcomes used in the large study is listed elsewhere (Berger et al., 2021). Here, we report the results related to PS, which was measured during the MOXO-CPT (NeuroTech Solutions Ltd., Jerusalem, Israel), a standardized computerized test designed to assess sustained attention deficits related to ADHD. Participants were seated at a quiet examination room at a distance of 60 cm from a computer screen (size:36 × 48 cm). Participants watched a demonstration video before performing the task for the first time. Furthermore, we made sure that participants understood the instructions and a research assistant was present throughout the entire session in the room. Participants performed the test five times during the study (see Figure 1A).

**FIGURE 2:**
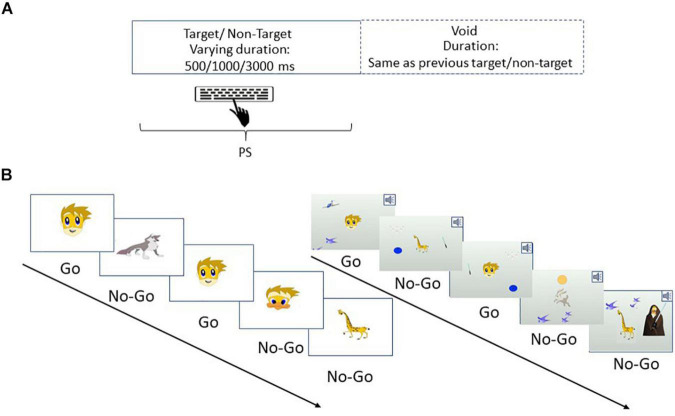
An example of the MOXO-CPT. **(A)** Stimuli are presented on the screen for varying amounts of time (500, 1000, or 3000 ms) followed by a **“void”** period of time of the same duration. Participants should respond to target stimuli as quickly as possible and withhold response to non-target stimuli. **(B)** PS was evaluated under several experimental conditions, in which distractor modality (none, visual, auditory, or combined) and distractor load (no load, low load, and high load) were manipulated. Left: an example of a task sequence with no distractors and no load. Right: an example of a task sequence with combined distractors and high load. All rights in the image are reserved to Neurotech Solutions Ltd.

The test consists of 8 levels, each containing 53 trials and lasts for 114.15 s. The total duration of the entire test is 15.2 min. On each trial, a stimulus (target or non-target) appears in the middle of the screen for a variable duration of time (0.5, 1, or 3 s) and is followed by a “void” of the same duration (Figure 2A). Of the 53 trials on each level, 33 included target stimuli and 20 contained non-target stimuli. Participants were instructed to respond to the target stimuli by clicking on the spacebar once, and only once, and withhold response to non-target stimuli.

Four performance indices are derived from each level of the MOXO-CPT (Cassuto et al., 2013; Berger and Cassuto, 2014; Berger et al., 2017; Peled et al., 2020). *PS* is the timing index of the task and is calculated as the total number of correct responses obtained while the target stimulus is still on the screen. Importantly, we distinguished between PS metric, which is operationalized as accurate responses performed in “good timing” (i.e., quick and correct responses to the target while the stimulus is still presented), and “attention” metric, which considers accurate but slow responses given during the void period.

#### MOXO-CPT Levels

Table 1 lists the various task levels in relation to distractor modality and load. The test consists of eight levels, each characterized by a different set of distractors, which differ in their modality (three options: auditory, visual, or both) and in their load (two options: low load—one distractor and high load—two distractors; see Figure 2B). A separate PS score was derived for each level, yielding eight different PS scores, as well as a total PS score (overall sum scores of PS index from all levels).

**TABLE 1:** The levels of the MOXO-CPT in terms of distractor modality and load.

Level #	Distractor modality		Distractor load	
		
	Visual	Auditory	Low (1)	High (2)
1	–	–	–	–
2	√	–	√	–
3	√	–	–	√
4	–	√	√	–
5	–	√	–	√
6	√	√	√	–
7	√	√	–	√
8	–	–	–	–

### Statistical Analyses

Treatment effects were analyzed using linear mixed effects models (LMMs), which are recommended for a study with a within-participant design, as they are able to account for correlations more optimally compared to ANOVA and automatically handle missing values, allowing maximum use of available data (Seltman, 2009). The LMM analysis was conducted using the R package nlme (Pinheiro et al., 2017) with maximized log-likelihood on the outcome measures, while subjects and level number were added as random factors. PS was examined immediately post-treatment (t1, N-t1) and at a 1 week follow up (t2, N-t2) for each stimulation type. We included stimulation type (tDCS + CT, tRNS + CT) and time (immediately after treatment, 1-week follow-up) as predictors. To better adjust for minor differences in the pre-treatment means, baseline performance was included as a covariate in the model: t0 baseline measures were used as baseline performance assessments for the first treatment received before crossover (tDCS + CT/tRNS + CT). To allow for an accurate assessment of measures after crossing over to the second treatment (tDCS + CT/tRNS + CT), we recalibrated the participants’ baseline measures using their latest assessment data from follow-up (t2), and set it as the new baseline for the second treatment after crossover (N-t0, see Figure 1; see also Looi et al., 2017; Clayton et al., 2019; Berger et al., 2021 for a similar statistical approach).

We verified that the residuals were normally distributed using a q-q plot and the Shapiro-Wilk normality test for all the measures. Measures were not normally distributed, we therefore applied the Tukey ladder of powers transformation as recommended (Tukey, 1977, see also Berger et al., 2021). Due to the exploratory nature of the study, the results reported here were not corrected for multiple comparisons. Still, we note that none of the results was significant at a α ≤ 0.05 after applying Bonferroni correction for multiple comparisons.

## Results

### The Effect of Treatment Type on Processing Speed With and Without Distractors

Our previous study showed a main effect of treatment type using the sum of PS scores across all eight levels of the MOXO-CPT (Berger et al., 2021). These results indicate greater improvement in PS following tRNS + CT compared with tDCS + CT, irrespective of the presence or absence of distractors or fatigue conditions.

To examine the effect of treatment type (tRNS + CT/tDCS + CT) on PS with and without environmental distractors, we predicted the post-treatment PS scores in each level for the two treatment conditions in two time points (post-treatment and follow up; t1 and t2, respectively), while covarying for the baseline PS scores at these levels. The results are summarized in Table 2. There was a significant effect of time, showing worsening in PS at follow up (t2) compared to post-treatment (t1) across all levels [β = −0.19 (SE = 0.07), *B* = −1.17 (SE = 0.42), *t*(396) = −2.78, *p* = 0.006, 95% CI (−0.3, −0.06)]. There was no significant effect of treatment [β = −0.19 (SE = 0.16), *B* = −1.2 (SE = 0.97), *t*(396) = −1.24, *p* = 0.21, 95% CI (−0.51, 0.11)]. However, the time × treatment interaction was significant [β = 0.25 (SE = 0.1), *B* = 1.5 (SE = 0.6), *t*(396) = 2.51, *p* = 0.012, 95% CI (0.05, 0.44)], indicating larger PS changes from post-treatment to follow-up following tRNS + CT compared to changes seen following tDCS + CT.

**TABLE 2:** A regression model of the MOXO-CPT PS total score post-treatment (t1) and at a 1-week follow-up (t2).

	B	Std error	DF	*t*-value	*P*-value
**Levels (1–8)**					
Intercept	0.23	0.23	396	1.03	0.3
PS (Baseline)	−0.07	0.04	396	−1.76	0.08
Treatment	−0.19	0.16	396	−1.24	0.21
Time	−0.19	0.07	396	−2.78	0.006*
Treatment × Time	0.25	0.1	396	2.51	0.013*

*p < 0.05, **p < 0.005, ***p < 0.0001.

### The Effect of Treatment Type on Processing Speed Given Cognitive Fatigue, Distractor Modality, and Distractor Load

#### Effects of Fatigue

To examine the effects of fatigue on PS, we defined a categorical predictor “fatigue,” which considers both task load and time spent on the task, with the assumption that high levels of load and blocks performed later during the task will lead to higher levels of cognitive fatigue (DeLuca, 2018). Since participants completed 2 repetitions without load (levels 1 and 8), 3 repetitions of the low load condition (levels 2, 4, and 6), and 3 repetitions of the high load condition (levels 3, 5, and 7; see Table 1), we incorporated these repetitions into the analyses when examining the effects of fatigue. Thus, the categorical predictor “fatigue” had three levels: Level 1 corresponded to the first repetition of each load type (levels 1–3 of the original task), Level 2 is the second level of low and high levels of load (levels 4 and 5), and Level 3 is the final levels of each load type (levels 6, 7, and 8). We predicted PS scores for the two treatment types in two time points, while covarying for the baseline PS scores at each level and controlling for fatigue (using the predictor “fatigue”).

The results are summarized in Table 3. There was a significant effect of fatigue [β = −0.18 (SE = 0.04), *B* = −1.1 (SE = 0.25), *t*(528) = −4.46, *p* = 0.00001, 95% CI (−0.26, −0.1)], indicating worse PS scores for higher levels of fatigue. In addition, there was a significant “fatigue” × treatment type interaction [β = −0.12 (SE = 0.06), *B* = 0.72 (SE = 0.35), *t*(528) = 2.07, *p* = 0.039, 95% CI (0.007, 0.23)], indicating better PS scores following tRNS + CT for high levels of fatigue compared to following tDCS + CT. All other effects were non-significant.

**TABLE 3:** A regression model of the MOXO-CPT PS scores in the presence of cognitive fatigue.

	B	Std error	DF	*t*-value	*P*-value
**Fatigue**					
Intercept	0.41	0.22	528	1.84	0.07
Baseline PS	−0.02	0.04	528	−0.46	0.65
Treatment type	−0.05	0.13	528	−0.43	0.67
Time	−0.07	0.05	528	−1.38	0.17
Fatigue	−0.18	0.04	528	−4.46	0.00001*
Fatigue × Treatment type	0.12	0.06	528	2.07	0.039*

*p < 0.05, **p < 0.005, ***p < 0.0001.

In a separate model, which examined fatigue in terms of time, we predicted PS scores for the two treatment types in two time points, while covarying for the baseline PS scores and controlling for fatigue. The results show a significant fatigue × time interaction [β = 0.13 (SE = 0.06), *B* = 0.8 (SE = 0.35), *t*(528) = 2.29, *p* = 0.02, 95% CI (0.02, 0.25)], indicating better performance at higher levels of fatigue at follow-up (t2) compared to post-treatment (t1; for further details see Supplementary Material 2).

Finally, due to *a priori* expectations for an estimated difference in PS at the first and last (eighth) levels of the task, we examined the effect of fatigue on PS by predicting PS scores for the eighth level of the task following each treatment type at two time points (t1 and t2), while covarying for the PS baseline scores from level 1, using both interactive and non-interactive models. A comparative model revealed a preference for the non-interactive model [ANOVA: *F*_(7,6)_ = 0.93; *p* = 0.34], which we report here. The results are summarized in Table 4. There was a main effect of treatment type, indicating greater improvement in PS following tRNS + CT compared to tDCS + CT [β = 0.34 (SE = 0.15), *B* = 2.06 (SE = 0.9), *t*(49) = 2.3, *p* = 0.03, 95% CI (0.05, 0.63)], and a main effect of time [β = 0.34 (SE = 0.15), *B* = 2.04 (SE = 0.9), *t*(49) = 2.25, *p* = 0.03, 95% CI (0.05, 0.64)], indicating further improvements in follow-up compared to post-treatment see Supplementary Figure 2.

**TABLE 4:** A regression model predicting PS scores at post-treatment (t1) and follow-up (t2) on level 8 of the MOXO-CPT, after covarying for PS on level 1.

	B	Std error	DF	*t*-value	*P*-value
**Fatigue non-interactive model**					
Intercept	−0.75	0.28	49	−2.7	0.01
Baseline PS–Level 1	0.39	0.11	49	3.54	0.001**
Treatment type	0.34	0.15	49	2.3	**0.03** *
Time	0.34	0.15	49	2.25	0.03*

*p < 0.05, **p < 0.005, ***p < 0.0001.

#### Effects of Distractor Load and Distractor Modality

The effects of distractor load and modality were predicted using the categorical predictors “LoadLevel” and “ModalityType,” respectively. “LoadLevel” was calculated based on the distractor load in each level of the MOXO-CPT, resulting in 3 levels: “No load” (no distractors; levels 1 and 8), “Low load” (one distractor; levels 2, 4, and 6), and “High load” (two distractors; levels 3, 5, and 7). “ModalityType” was calculated based on the modality type in each level: “no distractors” (levels 1 and 8), “visual” (levels 2 and 3), “auditory” (levels 4 and 5), and “combined” (both auditory and visual; levels 6 and 7). Using these newly defined variables, we examined the differences in PS under different distractor load and modality conditions by running two separate models, in which we predicted PS scores for all load or modality levels, respectively, at the two time points, while covarying for the baseline PS scores at each level.

As expected, there was a significant effect of load [β = −0.14(SE = 0.05), *B* = −0.84 (SE = 0.28), *t*(528) = −3.03, *p* = 0.003, 95% CI (−0.23, −0.05)] and of modality [β = −0.13 (SE = 0.03), *B* = −0.79 (SE = 0.2), *t*(528) = −4.12, *p* = 0.00001, 95% CI (−0.19, −0.07)], indicating worse performance for levels with higher load and with combined distractions compared to no load and lack of distractors. However, there were no significant interactions between distractor load and treatment type [β = 0.07 (SE = 0.06), *B* = 0.41 (SE = 0.39), *t*(528) = 1.04, *p* = 0.3, 95% CI (−0.06, 0.19)], nor between distractor modality and treatment type [β = 0.05 (SE = 0.04), *B* = 0.3 (SE = 0.27), *t*(528) = 1.1, *p* = 0.27, 95% CI (−0.04, 0.14)], indicating no differences in PS between the two treatment types under different distractor load or modalities. Finally, there were no significant effects of time nor of treatment (see Supplementary Material 2).

## Discussion

In the current study, we examined the effect of a combined treatment of tES (tDCS vs. tRNS) and CT on PS in a sample of unmedicated children diagnosed with ADHD. A previous analysis of the data showed that the average PS improves more following a 1-week intervention of tRNS + CT compared to tDCS + CT (Berger et al., 2021). Here, we show that this larger improvement in PS following tRNS + CT is seen in all task levels independently; However, this advantage of tRNS over tDCS is mainly evident for task conditions in which the level of cognitive fatigue is higher due to prolonged time in task, regardless of the nature of distractors or their load. Furthermore, the advantage of tRNS over tDCS was also seen in terms of side effects, subjects reported about less side effects (tingling, itching, etc.) following tRNS compared to tDCS, for further details see Berger et al., 2021.

The general preference for tRNS over tDCS, which is a main outcome of the current study, is in line with some recent reports involving healthy adult participants, which showed improvements in PS of a speeded attention task following tRNS compared to anodal tDCS or improvements in cognitive tasks compared to sham (Snowball et al., 2013; Popescu et al., 2016; Brevet-Aeby et al., 2019; Lema et al., 2021). Our current results further show long-term effects for tRNS + CT on PS for increased levels of cognitive fatigue, evident following 1 week of no treatment. The long-term enhancement of cognitive and brain functions following tRNS are consistent with those reported in other studies on various sensory (Herpich et al., 2019) and cognitive (Snowball et al., 2013) tasks. A recent study further showed PS improvements on a GNG task on the eighth day following three sessions of tRNS (Brevet-Aeby et al., 2019).

One potential account for the superiority of tRNS over tDCS seen in our study is the fact that both tES conditions were coupled with CT. While CT applied in isolation has shown only limited efficacy and transfer effects in training studies involving individuals with ADHD (see meta-analysis Cortese et al., 2015), it has been suggested the combination of tES with CT may be more effective in inducing longer term effects (Rubia, 2018; Berger et al., 2021). Combining CT with tES over prefrontal regions, which are known to underlie EF (Rubia, 2013), has been suggested to further enhance brain plasticity in ADHD compared to tES applied alone (Kuo and Nitsche, 2012; Rubia, 2018). These gains are potentially due to boosting training-induced plasticity through the addition of stimulation-induced plasticity (Ziemann and Siebner, 2008), which yields larger and long-lasting functional improvements that modify the impaired system (Cramer et al., 2011). A main finding in our study is that the preference for tRNS + CT over tDCS + CT is mainly evident in higher levels of cognitive fatigue. No other study, to the best of our knowledge, directly examined the effects of tRNS + CT on cognitive fatigue in pediatric ADHD. However, these findings are not entirely compatible with those from other studies which examined the effects of tRNS on cognitive fatigue in participants with multiple sclerosis (Palm et al., 2016; Salemi et al., 2019). Specifically, Palm et al. (2016) reported no effect of tRNS compared to sham over DLPFC on fatigue scores in subjects with multiple sclerosis, and Salemi et al. (2019) similarly reported no effect of tRNS applied to the primary motor cortex (M1) on cognitive fatigue compared to sham. This suggests that tRNS over M1 may only target the physical part of fatigue and other cortical areas might be tested to induce cognitive/psychosocial changes.

### The Lack of Effect for Distractor Load and Modality

While our study shows improved PS following tRNS + CT for higher levels of cognitive fatigue, we did not find a specific effect when controlling for distractor load or modality *per se*. This finding is in contradiction to our initial hypothesis, which was based on previous studies showing improvements in various cognitive tasks during more complex or demanding conditions (Popescu et al., 2016; Sheffield et al., 2020; Lema et al., 2021) following tRNS. In these cases, tRNS over the prefrontal cortices yielded better performance when task difficulty was manipulated (Popescu et al., 2016), or if participants exhibited more difficulties due to individual differences in cognitive abilities (Frank et al., 2018; Harty and Cohen Kadosh, 2019; Sheffield et al., 2020).

There are several accounts for this potential contradiction. First, none of the other studies used environmental distractors as load, as we did here. However, this type of load is particularly relevant to examine in children with ADHD, who have been shown to be strongly distracted by environmental distractions (Cassuto et al., 2013). In addition, all other studies used non-clinical samples of healthy adults with larger sample sizes. Therefore, it is possible that the small clinical pediatric population in our study and the use of a within-subjects design with a 1-week washout period concealed the potential effects of cognitive load or modality that we expected to find. The fact that we saw no such effect in our results calls for a potential replication with larger sample sizes of clinical populations.

### Effects of Transcranial Direct Current Stimulation

As for tDCS treatment, only one previous study examined the effects of tDCS on PS using the same MOXO-CPT in adults with ADHD (Jacoby and Lavidor, 2018). In this study, no changes in PS were found following a single session of tDCS only. While subjects underwent three sessions (baseline, active tDCS, and sham) and performed MOXO-CPT in a within-subject design, the authors emphasized the importance of possible learning effects following repetitions of MOXO-CPT; which may conceal the potential beneficial effects of the stimulation. Of note, other studies including adult populations also reported null effects on PS or even PS impairments following anodal tDCS in comparison to sham stimulation (Marshall et al., 2005; Adelhöfer et al., 2019).

The effects of tDCS on cognitive fatigue were examined in studies with either healthy adult populations or with a clinical sample (multiple sclerosis). While Borragán et al. (2018) reported no impact of anodal tDCS on cognitive fatigability-related PS in healthy adult subjects (Borragán et al., 2018), others suggested that anodal tDCS over DLPFC improves PS of cognitive tasks in healthy adult participants (Nelson et al., 2014; Sarasso et al., 2019) and in participants with multiple sclerosis (Hanken et al., 2016; Fiene et al., 2018), with the impact lasting for at least 6 h (McIntire et al., 2017). Given the small number of studies to date, and the heterogeneity of the methods applied and of the clinical profile of the patients, more studies are required in order to draw a more definitive conclusion (Saiote et al., 2014; Chalah et al., 2017; Lefaucheur et al., 2017; Hsu et al., 2021).

### Potential Mechanisms of Transcranial Electric Stimulation Action Under Fatigue Conditions

Processing speed and rapid cognitive processes are hypothesized to stem from specific activation patterns in prefrontal cortex areas, particularly the dorsolateral prefrontal cortex (DLPFC) (Takeuchi et al., 2011; Nouchi et al., 2020). It has been suggested that for individuals with slower PS, additional prefrontal regions are recruited in order to support successful task performance (Rypma et al., 2006; Takeuchi et al., 2011). However, the neural mechanisms underlying cognitive fatigue in relation to PS are still not fully understood. While it has been suggested that anodal stimulation over left DLPFC significantly attenuates the cognitive fatigue-related increase in PS in attention tasks for subjects with multiple sclerosis (Fiene et al., 2017), others suggested that counteracting the increase in PS may actually be achieved by targeting right parietal cortex (Hanken et al., 2016).

The exact neural mechanisms through which tRNS operates, nor the source of its advantage over tDCS, when both are combined with CT, are also not fully understood yet (Rufener et al., 2017). The most prevalent explanation for tRNS mode of action refers to a stochastic resonance mechanism, which describes the phenomenon of amplifying a weak signal to exceed a threshold by adding random noise to it (Stacey and Durand, 2000; Terney et al., 2008; Fertonani and Miniussi, 2017). Moreover, it was recently suggested that tRNS increases the synchronization of neural firing through the amplification of subthreshold oscillatory activity, which in turn reduces the amount of endogenous noise (Antal and Herrmann, 2016). Indeed, a recent study in juvenile mice (Sánchez-León et al., 2020) lends further support for the notion that tRNS induces neuro-plastic changes and alters cortical activity by improving signal-to-noise ratio at the neural level. In this study, excitatory effects associated with decreases in GABA levels were shown in the region directly beneath the electrodes, with no major histopathological alterations. The long-lasting effects of tRNS can be explained by the fact that changes in cognitive functions are accompanied by defined hemodynamic responses which are consistent with more efficient neurovascular activity coupling in specific brain regions (Snowball et al., 2013).

The superiority of tRNS applied over the left DLPFC and right IFG compared to anodal tDCS over the left DLPFC could reflect a negative effect of anodal tDCS on left DLPFC with respect to PS while tapping the same area by tRNS can affect positively. However, oversimplifying the idea that anodal tDCS and tRNS increase neuronal excitability and may consequently enhance behavioral performance (Paulus, 2011) could not explain the results at both neurophysiological and behavioral levels (Fertonani and Miniussi, 2017). In our previous study (Berger et al., 2021), we suggested three potential reasons that could explain the superiority of tRNS over tDCS.

Another potential account can refer to the nature of the MOXO-CPT, which is a complex GNG task which includes visual/auditory distractors at different load and fatigue conditions. This task can therefore be considered as a non-linear cognitive task which involves several brain regions (occipital, temporal, frontal, and motor networks) and hence has increased network demands, activating the neural attention system which requires synchronization of different brain regions (Lema et al., 2021). Considering the fact that adding noise to non-linear systems (which increase network demands) can actually increase performance (Storrs and Maiello, 2020) and that stochastic resonance has been shown to modulate intra- and inter-regional neural synchronization, may account for the superiority of tRNS over tDCS in improving PS in the MOXO-CPT. In other words, the requirement to ignore environmental distractions during the task necessitates synchronization of different brain regions, which increases neural network demands, and allows for additional neurons to tune in by the mechanism of stochastic resonance (Lema et al., 2021), thus increasing PS performance specifically following tRNS + CT. Future studies should further elucidate the potential mechanisms through which tRNS operates particularly in cases of cognitive fatigue during a complex cognitive task.

### Study Limitations and Future Directions

Despite its strengths, our study has a few limitations that should be noted. First, while our sample size is reasonable compared to previous studies in this population (e.g., Salehinejad et al., 2019), it is still rather small, providing only limited power which is required for finer-grained analyses (e.g., separation to subtypes of ADHD is not possible). In addition, participants underwent the MOXO-CPT five times during the trial, which may increase the likelihood of practice effects. Although our results did not reflect such a learning effect, but rather worsening of PS scores with time, a potential effect due to repeated exposure to the task cannot be ruled out. Furthermore, a 1-week “wash out” period between the two conditions in a cross-over design may still induce long-lasting effects of the first condition (see Berger et al., 2021). Finally, our study design compared two versions of tES, both combined with CT, which allowed for a comparison between the two types of tES. However, due to limited power, our design did not include other important controls, such as a sham + CT group or sham alone group, which may have provided a better understanding of the effect of each stimulation protocol or an assessment of the unique contribution of CT to PS changes. These limitations should be controlled for in future, carefully designed and powered studies. Further potential concern that highlights the need to include sham group in future studies is the fact that tDCS in contrast to sham may lead to impairment, rather than improvement in PS as reported in adult population; following anodal tDCS stimulation to DLPFC (Marshall et al., 2005), and also compared to tRNS as our results showed in pediatric ADHD (see Supplementary Materials 2.1).

Our findings have scientific, as well as potentially clinical implications for pediatric ADHD. The findings of the current study, as well as those of our previous study (Berger et al., 2021), support the superiority of tRNS + CT compared to tDCS + CT, making it a potentially viable treatment option for ADHD in children. The relatively short duration of treatment, along with its excellent safety profile, allow adding its translation to a potential standard-of-care that should be examined further carefully.

## Data Availability Statement

The raw data supporting the conclusions of this article will be made available by the authors, without undue reservation.

## Ethics Statement

The studies involving human participants were reviewed and approved by the Helsinki Committee of the Hebrew University and Hadassah Medical Center (Jerusalem, Israel). Written informed consent to participate in this study was provided by the participants’ legal guardian/next of kin.

## Author Contributions

OD-K collected data, analyzed the data, and wrote the draft of the manuscript. ESG and SB contributed to the data collection, psychological evaluations, and screening subjects. IB recruited and screened participants as a neurologist, contributed to conceptualization of the project, funding acquisition, and supervision and writing. RCK was in charge of project conceptualization, methodology, analysis, and supervision and writing. MN contributed to conceptualization of the project, methodology, writing, supervision, project administration, and funding acquisition. All authors reviewed and approved the final manuscript.

## Conflict of Interest

IB serves on the advisory board of Tech InnoSphere Engineering Ltd. RCK serves on the scientific advisory boards of Neuroelectrics Inc. and Tech InnoSphere Engineering Ltd. RCK filed a UK Patent *via* the University of Oxford for “Method for obtaining personalized parameters for transcranial stimulation, transcranial system, method of applying transcranial stimulation.” The remaining authors declare that the research was conducted in the absence of any commercial or financial relationships that could be construed as a potential conflict of interest.

## Publisher’s Note

All claims expressed in this article are solely those of the authors and do not necessarily represent those of their affiliated organizations, or those of the publisher, the editors and the reviewers. Any product that may be evaluated in this article, or claim that may be made by its manufacturer, is not guaranteed or endorsed by the publisher.

## References

[B1] Adalio C. J. Owens E. B. McBurnett K. Hinshaw S. P. Pfiffner L. J. (2018). Processing Speed Predicts Behavioral Treatment Outcomes in Children with Attention-Deficit/Hyperactivity Disorder Predominantly Inattentive Type. *J. Abnorm. Child Psychol.* 46 701–711. 10.1007/s10802-017-0336-z 28791531 PMC5807232

[B2] Adelhöfer N. Mückschel M. Teufert B. Ziemssen T. Beste C. (2019). Anodal tDCS affects neuromodulatory effects of the norepinephrine system on superior frontal theta activity during response inhibition. *Brain Struct. Funct.* 224 1291–1300. 10.1007/s00429-019-01839-3 30701308

[B3] Allenby C. Falcone M. Bernardo L. Wileyto E. P. Rostain A. Ramsay J. R. (2018). Transcranial direct current brain stimulation decreases impulsivity in ADHD. *Brain Stimul.* 11 974–981. 10.1016/j.brs.2018.04.016 29885858 PMC6109423

[B4] American Psychiatric Association (2013). *Diagnostic and Statistical Manual of Mental Disorders*, 5th Edn. Washington DC: APA Publishing.

[B5] Antal A. Herrmann C. S. (2016). Transcranial Alternating Current and Random Noise Stimulation: possible Mechanisms. *Neural Plast.* 2016:3616807 10.1155/2016/3616807 27242932 PMC4868897

[B6] Aron A. R. Robbins T. W. Poldrack R. A. (2004). Inhibition and the right inferior frontal cortex. *Trends Cogn. Sci*. 8 170–177. 10.1016/j.tics.2004.02.010 15050513

[B7] Ayache S. S. Chalah M. A. (2021). Cognitive fatigability in the healthy brain: neurophysiological substrates and the use of tDCS. *Clin. Neurophysiol.* 132 1714–1715. 10.1016/j.clinph.2021.03.018 33958264

[B8] Barkley R. A. DuPaul G. J. McMurray M. B. (1990). Comprehensive evaluation of attention deficit disorder with and without hyperactivity as defined by research criteria. *J. Consult. Clin. Psychol.* 58 775–789. 10.1037//0022-006x.58.6.775 2292627

[B9] Barkley R. A. Grodzinsky G. DuPaul G. J. (1992). Frontal lobe functions in attention deficit disorder with and without hyperactivity: a review and research report. *J. Abnorm. Child Psychol.* 20 163–188. 10.1007/BF00916547 1593025

[B10] Berger I. Cassuto H. (2014). The effect of environmental distractors incorporation into a CPT on sustained attention and ADHD diagnosis among adolescents. *J. Neurosci. Meth.* 222 62–68. 10.1016/j.jneumeth.2013.10.012 24211249

[B11] Berger I. Dakwar-Kawar O. Grossman E. S. Nahum M. Cohen Kadosh R. (2021). Scaffolding the attention-deficit/hyperactivity disorder brain using transcranial direct current and random noise stimulation: a randomized controlled trial. *Clin. Neurophysiol.* 132 699–707. 10.1016/j.clinph.2021.01.005 33561725

[B12] Berger I. Slobodin O. Cassuto H. (2017). Usefulness and Validity of Continuous Performance Tests in the Diagnosis of Attention-De fi cit Hyperactivity Disorder Children. *Arch. Clin. Neuropsychol.* 32 81–93. 10.1093/arclin/acw101 28122767

[B13] Bink M. van Nieuwenhuizen C. Popma A. Bongers I. L. van Boxtel G. J. (2014). Neurocognitive effects of neurofeedback in adolescents with ADHD: a randomized controlled trial. *J. Clin. Psychiatry* 75 535–542. 10.4088/JCP.13M08590 24922488

[B14] Bioulac S. Lallemand S. Rizzo A. Philip P. Fabrigoule C. Bouvard M. P. (2012). Impact of time on task on ADHD patient’s performances in a virtual classroom. *Eur. J. Paediatr. Neurol.* 16 514–521. 10.1016/J.EJPN.2012.01.006 22269913

[B15] Blakeman R. S. (2000). ADHD and distractibility: the role of distractor appeal. *Diss. Abstr. Int. B Sci. Eng.* 61:517.

[B16] Boksem M. A. Meijman T. F. Mm L. (2005). Effects of mental fatigue on attention: an ERP study. *Brain Res. Cogn. Brain Res.* 25 107–116. 10.1016/J.COGBRAINRES.2005.04.011 15913965

[B17] Borragán G. Gilson M. Guerrero-Mosquera C. Di Ricci E. Slama H. Peigneux P. (2018). Transcranial Direct Current Stimulation Does Not Counteract Cognitive Fatigue, but Induces Sleepiness and an Inter-Hemispheric Shift in Brain Oxygenation. *Front. Psychol.* 9:2351. 10.3389/FPSYG.2018.02351 30555378 PMC6284008

[B18] Brauer H. Breitling-Ziegler C. Moliadze V. Galling B. Prehn-Kristensen A. (2021). Transcranial direct current stimulation in attention-deficit/hyperactivity disorder: A meta-analysis of clinical efficacy outcomes. *Progress in Brain Research*, 264, 91–116. 10.1016/BS.PBR.2021.01.013 34167666

[B19] Brevet-Aeby C. Mondino M. Poulet E. Brunelin J. (2019). Three repeated sessions of transcranial random noise stimulation (tRNS) leads to long-term effects on reaction time in the Go/No Go task. *Neurophysiol. Clin.* 49 27–32. 10.1016/j.neucli.2018.10.066 30414823

[B20] Brown T. (2013). *A New Understanding of ADHD in Children and Adults: Executive Function Impairments.* New York: Routledge, 10.4324/9780203067536

[B21] Cassuto H. Ben-Simon A. Berger I. (2013). Using environmental distractors in the diagnosis of ADHD. *Front. Hum. Neurosci*. 7:805. 10.3389/fnhum.2013.00805 24319423 PMC3837230

[B22] Castellanos F. X. Lee P. P. Sharp W. Jeffries N. O. Greenstein D. K. Clasen L. S. (2002). Developmental trajectories of brain volume abnormalities in children and adolescents with attention-deficit/hyperactivity disorder. *Physiol. Behav.* 288 139–148. 10.1007/s11920-014-0498-0.Use 12365958

[B23] Castellanos F. X. Sonuga-Barke E. J. S. Scheres A. Di Martino A. Hyde C. Walters J. R. (2005). Varieties of attention-deficit/hyperactivity disorder-related intra-individual variability. *Biol. Psychiatry*. 57 1416–1423. 10.1016/j.biopsych.2004.12.005 15950016 PMC1236991

[B24] Chalah M. A. Riachi N. Ahdab R. Mhalla A. Abdellaoui M. Créange A. (2017). Effects of left DLPFC versus right PPC tDCS on multiple sclerosis fatigue. *J. Neurol. Sci.* 372 131–137. 10.1016/J.JNS.2016.11.015 28017199

[B25] Chhabildas N. Pennington B. F. Willcutt E. G. (2001). A comparison of the neuropsychological profiles of the DSM-IV subtypes of ADHD. *J. Abnorm. Child Psychol.* 29 529–540. 10.1023/A:1012281226028 11761286

[B26] Christakou A. Robbins T. W. Everitt B. J. (2004). Prefrontal Cortical-Ventral Striatal Interactions Involved in Affective Modulation of Attentional Performance: implications for Corticostriatal Circuit Function. *J. Neurosci.* 24 773–780. 10.1523/JNEUROSCI.0949-03.2004 14749421 PMC6729820

[B27] Clayton M. S. Yeung N. Kadosh R. C. (2019). Electrical stimulation of alpha oscillations stabilizes performance on visual attention tasks. *J. Exp. Psychol.* 148 203–220. 10.1037/xge0000502 30421943

[B28] Cook N. E. Braaten E. B. Surman C. B. H. (2018). Clinical and functional correlates of processing speed in pediatric Attention-Deficit/Hyperactivity Disorder: a systematic review and meta-analysis. *Child Neuropsychol.* 24 598–616. 10.1080/09297049.2017.1307952 28345402

[B29] Cortese S. Ferrin M. Brandeis D. Buitelaar J. Daley D. Dittmann R. W. (2015). Cognitive training for attention-deficit/hyperactivity disorder: meta-analysis of clinical and neuropsychological outcomes from randomized controlled trials. *J. Am. Acad. Child Adolesc. Psychiatry* 54 164–174. 10.1016/j.jaac.2014.12.010 25721181 PMC4382075

[B30] Cramer S. C. Sur M. Dobkin B. H. O’Brien C. Sanger T. D. Trojanowski J. Q. (2011). Harnessing neuroplasticity for clinical applications. *Brain* 134 1591–1609. 10.1093/brain/awr039 21482550 PMC3102236

[B31] Danielson M. L. Bitsko R. H. Ghandour R. M. Holbrook J. R. Kogan M. D. Blumberg S. J. (2018). Prevalence of Parent-Reported ADHD Diagnosis and Associated Treatment Among U.S. Children and Adolescents, 2016. *J. Clin. Child Adolesc. Psychol.* 47 199–212. 10.1080/15374416.2017.1417860 29363986 PMC5834391

[B32] de Oliveira Rosa V. Rosa Franco A. Abrahão Salum G. Moreira-Maia C. R. Wagner F. Simioni A. (2020). Effects of computerized cognitive training as add-on treatment to stimulants in ADHD: a pilot fMRI study. *Brain Imag. Behav.* 14 1933–1944. 10.1007/s11682-019-00137-0 31218531

[B33] DeLuca J. (2018). *Fatigue as a Window to the Brain.* Cambridge: MIT Press.

[B34] DuPaul G. J. Power T. J. Anastopoulos A. D. Reid R. (1998). *ADHD Rating Scale—IV: Checklists, Norms, and Clinical Interpretation.* New York: Guilford Press.

[B35] DuPaul G. J. Reid R. Anastopoulos A. D. Lambert M. C. Watkins M. W. Power T. J. (2016). Parent and teacher ratings of attention-deficit/hyperactivity disorder symptoms: factor structure and normative data. *Psychol. Assess*. 28 214–225. 10.1037/pas0000166 26011476

[B36] Elbaum T. Braw Y. Lev A. Rassovsky Y. (2020). Attention-Deficit/Hyperactivity Disorder (ADHD): integrating the MOXO-dCPT with an Eye Tracker Enhances Diagnostic Precision. *Sensors* 20:6386. 10.3390/S20216386 33182303 PMC7664925

[B37] Faundez V. De Toma I. Bardoni B. Bartesaghi R. Nizetic D. de la Torre R. (2018). Translating molecular advances in Down syndrome and Fragile X syndrome into therapies. *Eur. Neuropsychopharmacol.* 28 675–690. 10.1016/j.euroneuro.2018.03.006 29887288

[B38] Fertonani A. Miniussi C. (2017). Transcranial Electrical Stimulation: what We Know and Do Not Know About Mechanisms. *Neuroscientist* 23 109–123. 10.1177/1073858416631966 26873962 PMC5405830

[B39] Fiene M. Heinze H. J. Vielhaber S. Zaehle T. (2017). P316 Influence of transcranial direct current stimulation on electrophysiological and behavioral correlates of cognitive fatigue in multiple sclerosis. *Clin. Neurophysiol.* 128:e40. 10.1016/J.CLINPH.2016.10.198

[B40] Fiene M. Rufener K. S. Kuehne M. Matzke M. Heinze H. J. Zaehle T. (2018). Electrophysiological and behavioral effects of frontal transcranial direct current stimulation on cognitive fatigue in multiple sclerosis. *J. Neurol.* 265 607–617. 10.1007/S00415-018-8754-6 29356975

[B41] Frank B. Harty S. Kluge A. Cohen Kadosh R. (2018). Learning while multitasking: short and long-term benefits of brain stimulation. *Ergonomics* 61 1454–1463. 10.1080/00140139.2018.1563722 30587084

[B42] Fry A. F. Hale S. (2000). Relationships among processing speed, working memory, and fluid intelligence in children. *Biol. Psychol.* 54 1–34. 10.1016/S0301-0511(00)00051-X 11035218

[B43] Good P. I. (2006). *A Manager’s Guide to the Design and Conduct of Clinical Trials*, Second Edn. New York: wiley online library, 1–256.

[B44] Graziano P. A. Geffken G. R. Lall A. S. (2010). Heterogeneity in the Pharmacological Treatment of Children With ADHD. *Cogn. Behav. Soc. Funct. Diff.* 15 382–391. 10.1177/1087054710367772 20495162

[B45] Hanken K. Bosse M. Möhrke K. Eling P. Kastrup A. Antal A. (2016). Counteracting Fatigue in Multiple Sclerosis with Right Parietal Anodal Transcranial Direct Current Stimulation. *Front. Neurol.* 7:154. 10.3389/FNEUR.2016.00154 27708612 PMC5030283

[B46] Hartman C. A. Willcutt E. G. Rhee S. H. Pennington B. F. (2004). The relation between sluggish cognitive tempo and DSM-IV ADHD. *J. Abnorm. Child Psychol.* 32 491–503. 10.1023/B:JACP.0000037779.85211.29 15500029

[B47] Harty S. Cohen Kadosh R. (2019). Suboptimal Engagement of High-Level Cortical Regions Predicts Random-Noise-Related Gains in Sustained Attention. *Psychol. Sci.* 30 1318–1332. 10.1177/0956797619856658 31322974

[B48] Hedvall Å Fernell E. Holm A. Åsberg Johnels J. Gillberg C. Billstedt E. (2013). Autism, processing speed, and adaptive functioning in preschool children. *Sci. World J.* 2013:158263. 10.1155/2013/158263 23766675 PMC3673455

[B49] Herpich F. Melnick M. D. Agosta S. Huxlin K. R. Tadin D. Battelli L. (2019). Boosting learning efficacy with noninvasive brain stimulation in intact and brain-damaged humans. *J. Neurosci.* 39 5551–5561. 10.1523/JNEUROSCI.3248-18.2019 31133558 PMC6616291

[B50] Holtzer R. Shuman M. Mahoney J. R. Lipton R. Verghese J. (2011). Cognitive Fatigue Defined in the Context of Attention Networks. *Neuropsychol. Dev. Cogn.* 18:108. 10.1080/13825585.2010.517826 21128132 PMC3058923

[B51] Hsu W. Y. Cheng C. H. Zanto T. P. Gazzaley A. Bove R. M. (2021). Effects of Transcranial Direct Current Stimulation on Cognition, Mood, Pain, and Fatigue in Multiple Sclerosis: a Systematic Review and Meta-Analysis. *Front. Neurol.* 12:626113. 10.3389/FNEUR.2021.626113 33763014 PMC7982804

[B52] Jacoby N. Lavidor M. (2018). Null tDCS Effects in a Sustained Attention Task: the Modulating Role of Learning. *Front. Psychol.* 9:476. 10.3389/fpsyg.2018.00476 29681876 PMC5897507

[B53] Karam R. G. Breda V. Picon F. A. Rovaris D. L. Victor M. M. Salgado C. A. I. (2015). Persistence and remission of ADHD during adulthood: a 7-year clinical follow-up study. *Psychol. Med.* 45 2045–2056. 10.1017/S0033291714003183 25612927

[B54] Kaufman J. Birmaher B. Brent D. A. Ryan N. D. Rao U. (2000). K-SADS-PL. *J. Am. Acad. Child Adolesc. Psychiatry*. 39:1208. 10.1097/00004583-200010000-00002 11026169

[B55] Kessler S. K. Minhas P. Woods A. J. Rosen A. Gorman C. (2013). Dosage Considerations for Transcranial Direct Current Stimulation in Children: a Computational Modeling Study. *PLoS One* 8:e76112. 10.1371/journal.pone.0076112 24086698 PMC3785412

[B56] Kofler M. J. Rapport M. D. Sarver D. E. Raiker J. S. Orban S. A. Friedman L. M. (2013). Reaction time variability in ADHD: a meta-analytic review of 319 studies. *Clin. Psychol. Rev*. 33 795–811. 10.1016/j.cpr.2013.06.001 23872284

[B57] Kollins S. H. DeLoss D. J. Cañadas E. Lutz J. Findling R. L. Keefe R. S. E. (2020). A novel digital intervention for actively reducing severity of paediatric ADHD (STARS-ADHD): a randomised controlled trial. *Lancet Digit. Health* 2 e168–e178. 10.1016/S2589-7500(20)30017-0 33334505

[B58] Krause B. Cohen Kadosh R. (2013). Can transcranial electrical stimulation improve learning difficulties in atypical brain development? A future possibility for cognitive training. *Dev. Cogn. Neurosci*. 6 176–194. 10.1016/j.dcn.2013.04.001 23770059 PMC4064117

[B59] Krishnan C. Santos L. Peterson M. D. Ehinger M. (2015). Safety of Noninvasive Brain Stimulation in Children and Adolescents Chandramouli. *Physiol. Behav.* 176 139–148. 10.1016/j.brs.2014.10.012.Safety PMC4459719 25499471

[B60] Kuo M. F. Nitsche M. A. (2012). Effects of transcranial electrical stimulation on cognition. *Clin. EEG Neurosci.* 43 192–199. 10.1177/1550059412444975 22956647

[B61] Kuo M. F. Nitsche M. A. (2015). Exploring prefrontal cortex functions in healthy humans by transcranial electrical stimulation. *Neurosci. Bull*. 31 198–206. 10.1007/s12264-014-1501-9 25680572 PMC5563696

[B62] Lefaucheur J. P. Ma C. Mhalla A. Palm U. Ss A. Mylius V. (2017). The treatment of fatigue by non-invasive brain stimulation. *Clin. Neurophysiol.* 47 173–184. 10.1016/J.NEUCLI.2017.03.003 28410876

[B63] Lema A. Carvalho S. Fregni F. Gonçalves Ó. F. Leite J. (2021). The effects of direct current stimulation and random noise stimulation on attention networks. *Sci. Rep.* 11:6201. 10.1038/s41598-021-85749-7 33737661 PMC7973424

[B64] Looi C. Y. Lim J. Sella F. Lolliot S. Duta M. Avramenko A. A. (2017). Transcranial random noise stimulation and cognitive training to improve learning and cognition of the atypically developing brain: a pilot study. *Sci. Rep.* 7:4633. 10.1038/s41598-017-04649-x 28680099 PMC5498607

[B65] Marshall L. Mölle M. Siebner H. R. Born J. (2005). Bifrontal transcranial direct current stimulation slows reaction time in a working memory task. *BMC Neurosci.* 6:23. 10.1186/1471-2202-6-23 15819988 PMC1090588

[B66] Mayes S. D. Calhoun S. L. Chase G. A. Mink D. M. Stagg R. E. (2009). ADHD subtypes and co-occurring anxiety, depression, and oppositional-defiant disorder: differences in Gordon Diagnostic System and Wechsler working memory and processing speed index scores. *J. Atten. Disord.* 12 540–550. 10.1177/1087054708320402 18664713

[B67] McGee R. Brodeur D. Symons D. Andrade B. Fahie C. (2004). Time perception: does it distinguish ADHD and RD children in a clinical sample? *J. Abnorm. Child Psychol.* 32 481–490. 10.1023/B:JACP.0000037778.61929.1B 15500028

[B68] McIntire L. K. McKinley R. A. Nelson J. M. Goodyear C. (2017). Transcranial direct current stimulation versus caffeine as a fatigue countermeasure. *Brain Stimul.* 10 1070–1078. 10.1016/J.BRS.2017.08.005 28851554

[B69] Mizuno K. Tanaka M. Yamaguti K. Kajimoto O. Kuratsune H. Watanabe Y. (2011). Mental fatigue caused by prolonged cognitive load associated with sympathetic hyperactivity. *Behav. Brain Funct.* 7:17. 10.1186/1744-9081-7-17 21605411 PMC3113724

[B70] Moore A. L. Ledbetter C. (2019). The Promise of Clinician-Delivered Cognitive Training for Children Diagnosed with ADHD. *J. Mental Health Clin. Psychol.* 3 3–8.

[B71] Nejati V. Salehinejad M. A. Nitsche M. A. Najian A. Javadi A. H. (2017). Transcranial Direct Current Stimulation Improves Executive Dysfunctions in ADHD: implications for Inhibitory Control, Interference Control, Working Memory, and Cognitive Flexibility. *J. Atten. Disord.* 24 1928–1943. 10.1177/1087054717730611 28938852

[B72] Nelson J. T. Ra M. Golob E. J. Warm J. S. Parasuraman R. (2014). Enhancing vigilance in operators with prefrontal cortex transcranial direct current stimulation (tDCS). *NeuroImage* 85 909–917. 10.1016/J.NEUROIMAGE.2012.11.061 23235272

[B73] Nielsen N. P. Wiig E. H. (2011). AQT cognitive speed and processing efficiency differentiate adults with and without ADHD: a preliminary study. *Int. J. Psychiatry Clin. Pract.* 15 219–227. 10.3109/13651501.2011.582538 22121933

[B74] Nigg J. T. (2001). Is ADHD a disinhibitory disorder? *Psychol. Bull.* 127 571–598. 10.1037/0033-2909.127.5.571 11548968

[B75] Nouchi R. Kawata N. Y. D. S. Saito T. Himmelmeier R. M. Nakamura R. Nouchi H. (2020). Dorsolateral prefrontal cortex activity during a brain training game predicts cognitive improvements after four weeks’ brain training game intervention: evidence from a randomized controlled trial. *Brain Sci.* 10:560. 10.3390/brainsci10080560 32824185 PMC7464011

[B76] Palm U. Chalah M. A. Padberg F. Al-Ani T. Abdellaoui M. Sorel M. (2016). Effects of transcranial random noise stimulation (tRNS) on affect, pain and attention in multiple sclerosis. *Restor. Neurol. Neurosci.* 34 189–199. 10.3233/RNN-150557 26890095

[B77] Paulus W. (2011). Transcranial electrical stimulation (tES - tDCS; tRNS, tACS) methods. *Neuropsychol. Rehabil.* 21 602–617. 10.1080/09602011.2011.557292 21819181

[B78] Peled J. Cassuto H. Berger I. (2020). Processing speed as a marker to stimulant effect in clinical sample of children with high functioning autism spectrum disorder. *Nordic J. Psychiatry* 74 163–167. 10.1080/08039488.2019.1686063 31686565

[B79] Pelham W. E. Jr Waschbusch D. A. Hoza B. Gnagy E. M. Greiner A. R. Sams S. E. (2011). Music and video as distractors for boys with ADHD in the classroom: comparison with controls, individual differences, and medication effects. *J Abnorm Child Psychol.* 39 1085–1098. 10.1007/S10802-011-9529-Z 21695447

[B80] Pinheiro J. Bates D. DebRoy S. Sarkar D. R Core Team (2017). *nlme: Linear and Nonlinear Mixed Effects Models. R Package Version 3.1–130.* Available online at: https://CRAN.R-project.org/package=nlme

[B81] Polanía R. Nitsche M. A. Ruff C. C. (2018). Studying and modifying brain function with non-invasive brain stimulation. *Nat. Neurosci.* 21 174–187. 10.1038/s41593-017-0054-4 29311747

[B82] Popescu T. Krause B. Terhune D. B. Twose O. Page T. Humphreys G. (2016). Transcranial random noise stimulation mitigates increased difficulty in an arithmetic learning task. *Neuropsychologia* 81 255–264. 10.1016/j.neuropsychologia.2015.12.028 26731199 PMC4749538

[B83] Reed T. Cohen Kadosh R. (2018). Transcranial electrical stimulation (tES) mechanisms and its effects on cortical excitability and connectivity. *J Inherit. Metab. Dis.* 41 1123–1130. 10.1007/s10545-018-0181-4 30006770 PMC6326965

[B84] Rommelse N. Luman M. Kievit R. (2020). Slow processing speed: a cross-disorder phenomenon with significant clinical value, and in need of further methodological scrutiny. *Eur. Child Adolesc. Psychiatry* 29 1325–1327. 10.1007/S00787-020-01639-9 32915272

[B85] Rubia K. (2013). Functional brain imaging across development. *Eur. Child Adolesc. Psychiatry* 22 719–731. 10.1007/S00787-012-0291-8 22729957 PMC3853580

[B86] Rubia K. (2018). Cognitive Neuroscience of Attention Deficit Hyperactivity Disorder (ADHD) and Its Clinical Translation. *Front. Hum. Neurosci.* 12:100. 10.3389/fnhum.2018.00100 29651240 PMC5884954

[B87] Rubia K. Oosterlaan J. Sergeant J. A. Brandeis D. Leeuwen V. (1998). Inhibitory dysfunction in hyperactive boys. *Behav. Brain Res.* 94 25–32. 10.1016/S0166-4328(97)00166-6 9708836

[B88] Rucklidge J. J. Tannock R. (2002). Neuropsychological profiles of adolescent with ADHD: effects of reading difficulties and gender. *J Child Psychol Psychiatry* 43 988–1003. 10.1111/1469-7610.00227 12455921

[B89] Rufener K. S. Ruhnau P. Heinze H. J. Zaehle T. (2017). Transcranial random noise stimulation (tRNS) shapes the processing of rapidly changing auditory information. *Front. Cell. Neurosci.* 11:162. 10.3389/fncel.2017.00162 28642686 PMC5463504

[B90] Rypma B. Berger J. S. Prabhakaran V. Martin Bly B. Kimberg D. Y. Biswal B. B. (2006). Neural correlates of cognitive efficiency. *NeuroImage* 33 969–979. 10.1016/j.neuroimage.2006.05.065 17010646

[B91] Saiote C. Goldschmidt T. Timäus C. Steenwijk M. D. Opitz A. Antal A. (2014). Impact of transcranial direct current stimulation on fatigue in multiple sclerosis. *Restor. Neurol. Neurosci.* 32 423–436. 10.3233/RNN-130372 24531295

[B92] Salehinejad M. A. Wischnewski M. Nejati V. Vicario C. M. Nitsche M. A. (2019). Transcranial direct current stimulation in attention-deficit hyperactivity disorder: a meta-analysis of neuropsychological deficits. *PLoS One* 14:e0215095. 10.1371/journal.pone.0215095 30978259 PMC6461252

[B93] Salemi G. Vazzoler G. Ragonese P. Bianchi A. Cosentino G. Croce G. F. B. (2019). Application of tRNS to improve multiple sclerosis fatigue: a pilot, single-blind, sham-controlled study. *J. Neural Transm.* 126 795–799. 10.1007/S00702-019-02006-Y 31054015

[B94] Sánchez-León C. A. Sánchez-López Á. Gómez-Climent M. A. Cordones I. Kadosh R. C. Márquez-Ruiz J. (2020). Impact of chronic transcranial Random-Noise Stimulation (tRNS) on prefrontal cortex excitation-inhibition balance in juvenile mice. *BioRxiv* [Preprint]. 10.1101/2020.09.04.282889 34167661

[B95] Santarnecchi E. Brem A. K. Levenbaum E. Thompson T. Kadosh R. C. Pascual-Leone A. (2015). Enhancing cognition using transcranial electrical stimulation. *Curr. Opinion Behav. Sci.* 4 171–178. 10.1016/j.cobeha.2015.06.003

[B96] Sarasso P. Ninghetto M. Salatino A. Ronga I. Bongiardina A. Iarrobino I. (2019). Everything is (still) illuminated: dual right cathodal-left anodal tDCS of PPC prevents fatigue on a visual detection task. *Brain Stimul.* 12 187–189. 10.1016/J.BRS.2018.09.017 30314901

[B97] Scheres A. Oosterlaan J. Sergeant J. A. (2001). Response inhibition in children with DSM-IV subtypes of AD/HD and related disruptive disorders: the role of reward. *Child Neuropsychol.* 7 172–189. 10.1076/chin.7.3.172.8746 12187474

[B98] Seltman H. J. (2009). “Mixed models. A flexible approach to correlated data,” in *Experimental Design and Analysis* (Pittsburgh, PA: Carnegie Mellon University).

[B99] Sergeant J. A. (2005). Modeling attention-deficit/hyperactivity disorder: a critical appraisal of the cognitive-energetic model. *Biol. Psychiatry*. 57 1248–1255. 10.1016/j.biopsych.2004.09.010 15949995

[B100] Shanahan M. A. Pennington B. F. Yerys B. E. Scott A. Boada R. Willcutt E. G. (2006). Processing speed deficits in attention deficit/hyperactivity disorder and reading disability. *J Abnorm. Child Psychol.* 34 585–602. 10.1007/s10802-006-9037-8 16850284

[B101] Sheffield J. G. Raz G. Sella F. Kadosh R. C. (2020). How can noise alter neurophysiology in order to improve human behaviour? A combined transcranial random noise stimulation and electroencephalography study. *BioRxiv* [preprint]. 10.1101/2020.01.09.900118

[B102] Sherwell S. Reid S. M. Reddihough D. S. Wrennall J. Ong B. Stargatt R. (2014). Measuring intellectual ability in children with cerebral palsy: can we do better? *Res. Dev. Disabil.* 35 2558–2567. 10.1016/J.RIDD.2014.06.019 25005064

[B103] Slobodin O. Yahav I. Berger I. (2020). A Machine-Based Prediction Model of ADHD Using CPT Data. *Front. Hum. Neurosci.* 14:560021. 10.3389/fnhum.2020.560021 33093829 PMC7528635

[B104] Slusarek M. Velling S. Bunk D. Eggers C. (2001). Motivational effects on inhibitory control in children with ADHD. *J. Am. Acad. Child Adolesc. Psychiatry* 40 355–363. 10.1097/00004583-200103000-00016 11288778

[B105] Snowball A. Tachtsidis I. Popescu T. Thompson J. Delazer M. Zamarian L. (2013). Long-term enhancement of brain function and cognition using cognitive training and brain stimulation. *Curr. Biol.* 23 987–992. 10.1016/j.cub.2013.04.045 23684971 PMC3675670

[B106] Soltaninejad Z. Nejati V. Ekhtiari H. (2015). Effect of Anodal and Cathodal Transcranial Direct Current Stimulation on DLPFC on Modulation of Inhibitory Control in ADHD. *J. Atten. Disord.* 23 325–332. 10.1177/1087054715618792 26689935

[B107] Sotnikova A. Soff C. Tagliazucchi E. Becker K. (2017). Transcranial Direct Current Stimulation Modulates Neuronal Networks in Attention Deficit Hyperactivity Disorder. *Brain Topogr.* 30 656–672. 10.1007/s10548-017-0552-4 28213645

[B108] Stacey W. C. Durand D. M. (2000). Stochastic resonance improves signal detection in hippocampal CA1 neurons. *J. Neurophysiol.* 83 1394–1402. 10.1152/JN.2000.83.3.1394 10712466

[B109] Storrs K. R. Maiello G. (2020). A Model for Neural Network Modeling in Neuroscience. *J. Neurosci.* 40 7010–7012. 10.1523/JNEUROSCI.1205-20.2020 32907932 PMC7480246

[B110] Takeuchi H. Taki Y. Hashizume H. Sassa Y. Nagase T. Nouchi R. (2011). Effects of training of processing speed on neural systems. *J. Neurosci.* 31 12139–12148. 10.1523/JNEUROSCI.2948-11.2011 21865456 PMC6623215

[B111] Terney D. Chaieb L. Moliadze V. Antal A. Paulus W. (2008). Increasing Human Brain Excitability by Transcranial High-Frequency Random Noise Stimulation. *J. Neurosci.* 28 14147–14155. 10.1523/JNEUROSCI.4248-08.2008 19109497 PMC6671476

[B112] Thunberg C. Messel M. S. Raud L. Huster R. J. (2020). tDCS over the inferior frontal gyri and visual cortices did not improve response inhibition. *Sci. Rep.* 10:7749. 10.1038/s41598-020-62921-z 32385323 PMC7210274

[B113] Tukey J. (1977). *Exploratory Data Analysis.* Boston: Addison-Wesley Pub.Co.

[B114] van der Meere J. Stemerdink N. Gunning B. (1995). Effects of presentation rate of stimuli on response inhibition in ADHD children with and without tics. *Percept. Mot. Skills* 81 259–262. 10.2466/pms.1995.81.1.259 8532467

[B115] Visser S. N. Danielson M. L. Bitsko R. H. Holbrook J. R. Kogan M. D. Ghandour R. M. (2014). Trends in the parent-report of health care provider-diagnosed and medicated attention-deficit/hyperactivity disorder: United States, 2003-2011. *J. Am. Acad. Child Adolesc. Psychiatry* 53 34–46. 10.1016/j.jaac.2013.09.001 24342384 PMC4473855

[B116] Weigard A. Huang-Pollock C. (2017). The role of speed in ADHD-related working memory deficits: a time-based resource-sharing and diffusion model account. *Physiol. Behav.* 176 139–148. 10.1177/2167702616668320 28533945 PMC5437983

[B117] Weiler M. D. Bernstein J. H. Bellinger D. C. Waber D. P. (2001). Processing speed in children with Attention Deficit/Hyperactivity Disorder, inattentive type. *Child Neuropsychol.* 6 218–234. 10.1076/chin.6.3.218.3156 11402399

[B118] Westwood S. J. Radua J. Rubia K. (2021). Noninvasive brain stimulation in children and adults with attention-deficit/hyperactivity disorder: a systematic review and meta-analysis. *J. Psychiatry Neurosci.* 46 E14–E33. 10.1503/jpn.190179 33009906 PMC7955851

[B119] Wexler B. E. Iseli M. Leon S. Zaggle W. Rush C. Goodman A. (2016). Cognitive Priming and Cognitive Training: immediate and Far Transfer to Academic Skills in Children. *Sci. Rep.* 6:32859. 10.1038/srep32859 27615029 PMC5018694

[B120] Willcutt E. G. Doyle A. E. Nigg J. T. Faraone S. V. Pennington B. F. (2005). Validity of the executive function theory of attention-deficit/hyperactivity disorder: a meta-analytic review. *Biol. Psychiatry* 57 1336–1346. 10.1016/j.biopsych.2005.02.006 15950006

[B121] Zhao H. Qiao L. Fan D. Zhang S. Turel O. Li Y. (2017). Modulation of brain activity with noninvasive transcranial direct current stimulation (tDCS): clinical applications and safety concerns. *Front. Psychol.* 8:685. 10.3389/fpsyg.2017.00685 28539894 PMC5423956

[B122] Ziemann U. Siebner H. R. (2008). Modifying motor learning through gating and homeostatic metaplasticity. *Brain Stimul*. 1 60–66. 10.1016/j.brs.2007.08.003 20633369

