# Supplementary material for: Examining the Effect of Transcranial Electrical Stimulation and Cognitive Training on Processing Speed in Pediatric Attention Deficit Hyperactivity Disorder: A Pilot Study
Source: Front Hum Neurosci. 2022 Jul 27;16:791478. doi: 10.3389/fnhum.2022.791478 (PMC9363890; doi:10.3389/fnhum.2022.791478)
Supplement: Supplementary file 1 [file Data_Sheet_1.docx]

Supplementary Material

# Supplementary Data Including:

# Supplemental material 1: one supplementary figure

Supplemental material 2: 3 supplementary tables and one supplementary figure

# Supplemental material 1


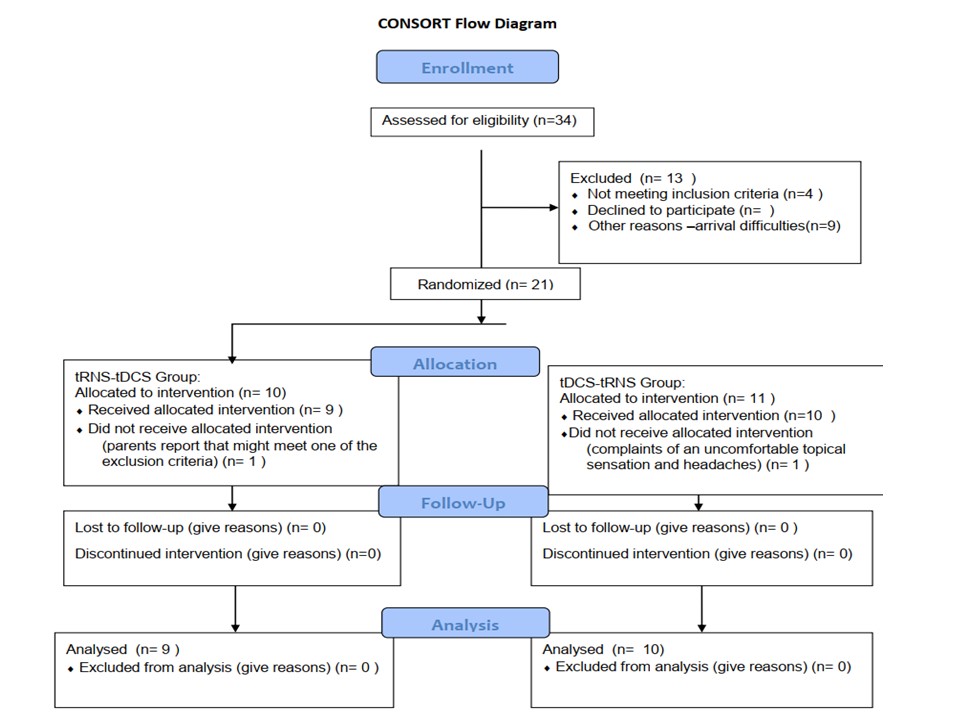


**Supplemental Figure S1.** Consort flow diagram of the randomized cross-over study.

# Supplemental material 2

### Effect of treatment type on PS in the presence of cognitive fatigue, in terms of time

In order to examine changes in PS scores under fatigue conditions in terms of time, we predicted the PS scores for the two treatment types in two time points (t1 and t2), while covarying for the baseline PS scores at each level and controlling for fatigue. There was a main effect of treatment [β=.19 (SE=.05), B=1.12 (SE=.03), t(528)=3.64, p=.0003), 95% CI (.09, .29)]], indicating better performance following tRNS+CT compared to tDCS+CT, see **Table S1, Figure S2.1**. Furthermore, there was a main effect of time [β=-.33 (SE=.13), B=-2.03 (SE=.77), t(528)=-2.66, p=.008), 95% CI (-.58, -.09)], and a main effect of fatigue [β=-.32 (SE=.09), B=-1.96 (SE=.56), t(528)=-3.5, p=.0005), 95% CI (-.5,-.14)], indicating significant worsening at follow up (t2) compared to post treatment (t1) and significant worsening in performance at higher fatigue levels, respectively.

**Table S1**. A regression model of the MOXO-CPT PS scores post-treatment (t1) and at follow-up one week later (t2) in the presence of cognitive fatigue.

|  | **Β** | **Std Error** | **DF** | **t-value** | **p-value** |
| --- | --- | --- | --- | --- | --- |
| **Fatigue** | | | | | |
| Intercept | .7 | .28 | 528 | 2.49 | .01 |
| PS (Baseline) | -.03 | .04 | 528 | -.68 | .5 |
| Treatment | .19 | .05 | 528 | 3.64 | .**0003**** |
| Time | -.33 | .13 | 528 | -2.66 | **.008*** |
| Fatigue | -.32 | .09 | 528 | -3.5 | **.0005**** |
| Fatigue X Time | .13 | .06 | 528 | 2.29 | .**02*** |

*p<.05; **p<0.005; ***p<0.0001


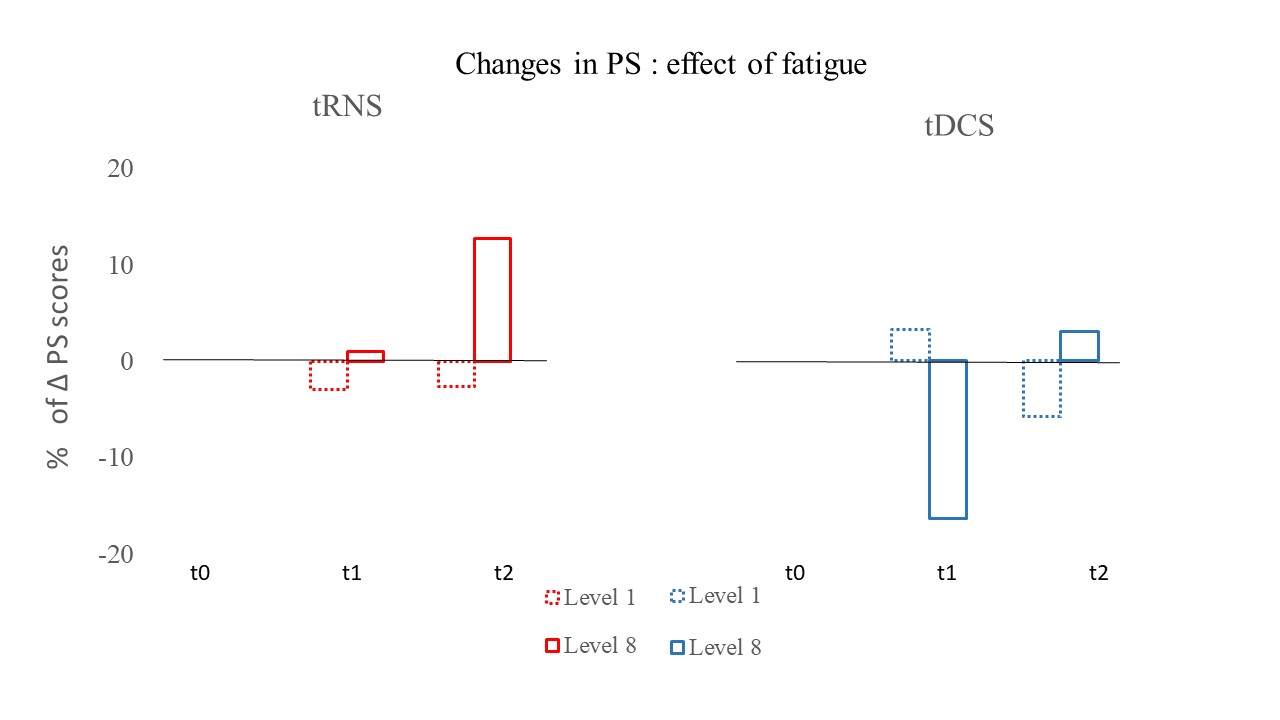


**Figure S2.1**. Percentage of changes in PS scores following tRNS + CT (left) and tDCS + CT (right) in both levels 1 and 8. Percentage of changes were calculated based on each previous t.

### 2.2 Effect of treatment type on PS in the presence of distractor load and under distractor modality

**Table S2**. A regression model of the MOXO-CPT PS scores post-treatment (t1) and at follow-up one week later (t2) in the presence of cognitive load.

|  | **Β** | **Std Error** | **DF** | **t-value** | **p-value** |
| --- | --- | --- | --- | --- | --- |
| **Distractor load** | | | | | |
| Intercept | .34 | .23 | 528 | 1.48 | .14 |
| PS (Baseline) | -.03 | .04 | 528 | -.76 | .45 |
| Treatment | .04 | .15 | 528 | .29 | .77 |
| Time | -.07 | .05 | 528 | -1.37 | .17 |
| Load | -.14 | .05 | 528 | -3.03 | **.003**** |
| Load X Treatment | .07 | .06 | 528 | 1.04 | .3 |

*p<.05; **p<0.005; ***p<0.0001

**Table S3**. A regression model of the MOXO-CPT PS scores post-treatment (t1) and at follow-up one week later (t2) in the presence of distractors at different modalities.

|  | **Β** | **Std Error** | **DF** | **t-value** | **p-value** |
| --- | --- | --- | --- | --- | --- |
| **Distractor modality** | | | | | |
| Intercept | .37 | .22 | 528 | 1.66 | .1 |
| PS (Baseline) | -.04 | .04 | 528 | -.91 | .36 |
| Treatment | .06 | .12 | 528 | .51 | .61 |
| Time | -.07 | .05 | 528 | -1.38 | .17 |
| Modality | -.13 | .03 | 528 | -4.12 | **.00001***** |
| Modality X Treatment | .05 | .04 | 528 | 1.1 | .27 |

*p<.05; **p<0.005; ***p<0.0001
